# Practical implications of erythromycin resistance gene diversity on surveillance and monitoring of resistance

**DOI:** 10.1093/femsec/fiy006

**Published:** 2018-01-15

**Authors:** Jinlyung Choi, Elizabeth L Rieke, Thomas B Moorman, Michelle L Soupir, Heather K Allen, Schuyler D Smith, Adina Howe

**Affiliations:** 1 Department of Agricultural and Biosystems Engineering, Iowa State University, 1201 Sukup Hall, Ames, IA 50011, USA; 2 National Laboratory for Agriculture and the Environment, USDA-ARS, 2110 University Blvd, Ames, IA 50011, USA; 3 Food Safety and Enteric Pathogens Research Unit, National Animal Disease Center, USDA-ARS, 1920 Dayton Ave, Ames, IA, 50010, USA; 4 Department of Bioinformatics and Computational Biology, Iowa State University, 2014 Molecular Biology Building, Ames, IA 50011, USA

**Keywords:** antibiotic, resistance, target, environment, surveillance, genes

## Abstract

Use of antibiotics in human and animal medicine has applied selective pressure for the global dissemination of antibiotic-resistant bacteria. Therefore, it is of interest to develop strategies to mitigate the continued amplification and transmission of resistance genes in environmental reservoirs such as farms, hospitals and watersheds. However, the efficacy of mitigation strategies is difficult to evaluate because it is unclear which resistance genes are important to monitor, and which primers to use to detect those genes. Here, we evaluated the diversity of one type of macrolide antibiotic resistance gene (*erm*) in one type of environment (manure) to determine which primers would be most informative to use in a mitigation study of that environment. We analyzed all known *erm* genes and assessed the ability of previously published *erm* primers to detect the diversity. The results showed that all known *erm* resistance genes group into 66 clusters, and 25 of these clusters (40%) can be targeted with primers found in the literature. These primers can target 74%–85% of the *erm* gene diversity in the manures analyzed.

## Teaser

We evaluate and identify the most effective primer gene targets to detect erythromycin resistance originating from manure sources.

## INTRODUCTION

Antibiotic resistance is a global challenge, with increasing resistance to antibiotics threatening our ability to treat both human and animal diseases (WHO). Antibiotic use in human medicine and animal agriculture has increased environmental reservoirs of antibiotic resistance genes, which in turn has increased the risk of transmission of antibiotic-resistant bacteria to both humans and animals (McEwen and Fedorka-Cray 2002; Vaz-Moreira *et al.*^2014^). This linkage has resulted in the prioritization of understanding how resistance moves from environmental sources to clinical pathogens and the associated influence of human activity. To understand the movement of antibiotic resistance in the environment, we need accessible tools that can provide large-scale surveillance of resistance in diverse environmental samples.

Molecular microbiology advances have allowed us to leverage amplification and subsequent sequencing of DNA that encodes for antibiotic resistance genes, resulting in our awareness of an incredibly diverse global reservoir of environmental “resistomes”. Generally, metagenomic shotgun sequencing is a costly tool for antibiotic gene surveillance as it provides information on ‘all’ genes in an environmental sample. Among these genes, only a fraction (0.01%–1%) are related to antibiotic resistance, resulting in a significant majority of sequences from metagenomes not readily usable for resistance detection (Shi *et al.*^2013^; Li *et al.*^2015^). A promising alternative to metagenomic sequencing is high-throughput amplicon qPCR assays, such as the Wafergen Smartchip that has been previously used for several resistance surveillance studies (Shi *et al.*^2013^; Wang *et al.*^2014^; Karkman *et al.*^2016^; Muziasari *et al.*^2016^; Stedtfeld *et al.*^2017^). Unlike the broad scope of metagenomic sequencing, high-throughput qPCR assays target a suite of genes using primers and can quantify hundreds of targeted resistance genes and multiple samples simultaneously (e.g. one Wafergen Smartchip contains 5184 assays). Consequently, the price per gene or sample of these assays for resistance gene detection is orders of magnitude less than metagenomic sequencing, making it more conducive to large-scale surveillance. A significant limitation of this technology is the need to develop primer-based assays for each targeted gene of interest that are effective for high-throughput amplification conditions.

We are increasingly aware that certain genes may be more related to the risks of the emergence or persistence of resistance than others. For example, integrons and sulfonamide resistance genes have been used to detect anthropogenic contaminants (Wang *et al.*^2014^; Gillings *et al.*^2015^). Further, specific environments (mammalian gut, manure, wastewater, etc.) have been observed to be enriched in antibiotic resistance genes relative to soil or water environments (Chee-Sanford *et al.*^2009^; Koike *et al.*^2010^; Garder, Moorman and Soupir 2014; Joy *et al.*^2014^; Luby, Moorman and Soupir 2016), suggesting that these environments are potential reservoirs of resistance genes. Among the hundreds of genes associated with antibiotic resistance that are observed in environmental metagenomes, selecting the key targets relevant to the spread of resistance is a significant and important opportunity. In this study, we demonstrate how we have chosen specific genes that are the most effective among previously targeted genes to serve as indicators for antibiotic resistance and to understand resistance hotspots and transmission. This framework, while developed for agriculturally impacted environments, can be broadly applied to the selection of genes from varying resistance gene classes and environments. Specifically, this effort focuses on understanding the diversity of erythromycin ribosomal methylase (*erm*) gene and the most relevant gene targets for understanding the spread of *erm-*associated resistance from manure sources to the environment.

*Erm* genes encode resistance to macrolide antibiotics, which have long been used to treat Gram-positive and certain Gram-negative pathogens infecting humans, swine and cattle (Roberts *et al.*^2008^; Pyörälä *et al.*^2014^). Broadly, macrolide antibiotics act by binding to the 23S subunit of the bacterial ribosome, causing premature release of peptides during translation. The *erm* genes cause resistance by methylating rRNA at the active site, reducing the ability of macrolide antibiotics to bind to the ribosome (Weisblum 1998; Vester and Douthwaite 2001). *Erm*-mediated resistance to macrolides has also been observed to confer resistance against other antibiotics, including lincosamide and streptogramin B (MLS_B_ resistance) (Leclercq and Courvalin 1991). The widespread use of macrolides and their relevance for both animal and human health has resulted in a research emphasis on *erm* genes and their bacterial hosts as key targets for understanding the development of resistance and its spread in agricultural environments. Previously, *erm* genes have been detected in various agricultural settings, including swine manure, lagoon water, soils, surface and subsurface drainage from fields, and groundwater surrounding and underlying animal production facilities (Chen *et al.*^2007^; Knapp *et al.*^2010^; Koike *et al.*^2010^; Joy *et al.*^2013^, ^2014^; Whitehead and Cotta 2013; Fahrenfeld *et al.*^2014^; Garder, Moorman and Soupir 2014; Soni *et al.*^2015^; Luby, Moorman and Soupir 2016).

Most of our previous knowledge of *erm* genes and their associated amplicon targets have stemmed from the characterization and sequencing of bacterial isolates and their phenotypic resistance to MLS_B_ antibiotics (Pyörälä *et al.*^2014^). A total of 21 unique classes of *erm* genes have been identified based on sequence homology to protein-coding *erm* sequences from cultured bacteria (Roberts *et al.*^1999^). More recently, metagenomic analyses of DNA from the total microbial community in environmental samples has expanded what is known about *erm* diversity beyond these 21 classes, showing that the *erm* class of genes is comprised of numerous sequence variants from diverse bacterial hosts (Fang *et al.*^2015^; Li *et al.*^2015^). These sequence variants are present in a range of abundances depending on their environment of origin. The focus of this study was to better understand the diversity of *erm* genes and to target the gene variants that could be indicative of resistance originating from manure and spreading to agricultural soil and water environments.

## MATERIALS AND METHODS

### Phylogenetic analysis of erm genes

Gene sequencing sharing high similarity to *ermA, ermB, ermC* and *ermF* were obtained from publicly available databases. The Ribosomal Database Project Fungene Repository (Fish *et al.*^2013^) was used to obtain *ermB*- and *ermC*-associated sequences. It was required that sequences share 97% amino acid sequence coverage to established HMM protein models for Fungene gene families “Resfam_ermA”, “Resfam_ermB” and “Resfam_ermC” (Version 8.8). Additionally, *ermF* gene nucleotide sequences were obtained from proteins listed in the ARDB-Antibiotic Resistance Genes Database (version 1.1, July 3, 2009) (Liu and Pop 2009) and associated with the annotation “*ermF*”. All *erm*-associated sequences were combined and clustered at 99% nucleotide similarity using CD-HIT (v4.6.1c) (Li and Godzik 2006; Fu *et al.*^2012^), resulting in 66 unique clusters. One representative sequence for each cluster was identified by CD-HIT and was aligned using Muscle (v3.8.31) (Edgar 2004) with the following parameters: gap open –400, gap extend 0, clustering method UPGMB. A maximum-likelihood phylogenetic tree was constructed from this alignment using FastTree (v2.1.8) (Price, Dehal and Arkin 2010) with default parameters. Taxonomy was identified based on annotations in the NCBI non-redundant nucleotide database (NCBI Resource Coordinators 2017).

To consider an *erm* gene sequence to be associated with a previously targeted PCR primer sequence, both forward and reverse primers were required to share 100% nucleotide similarity over a minimum of 17 bp of the primer length.

### Manure metagenomic datasets

The presence of *erm* genes was characterized in swine and cattle manures. For swine manure, DNA was extracted from two biological replicates (three technical replicates each) of swine manure originating from Iowa State University's Northeast Research and Demonstration Farm, near Nashua, IA (43.0° N, 92.5° W). Metagenomic libraries were prepared and sequenced at Iowa State University DNA Sequencing Facility on a HiSeq 2500 instrument (Illumina, San Diego, CA) according to manufacturer's instructions. These datasets are deposited in the NCBI SRA as project SRP109083 (Table S1, Supporting Information). Sequences were compared to representatives of *erm* genes described above (BLAST, v2.4.0+) (Camacho *et al.*^2009^). Sequences were annotated as *erm* genes if they matched the representative sequence within a cluster with a minimum e-value of 1e-5 and if both paired-end reads matched the same representative target. The abundance of *erm* sequences in each sample was calculated as the total number of reads meeting these criteria.

Cattle manure metagenomes were obtained from a previously published study of antibiotic resistant genes in commercial cattle as they moved through the process of beef production from feedlot entry to slaughter (Noyes *et al.*^2016^). The presence of *erm* sequences in these samples was determined by the total number of reads that shared sequence homology (BLAST, v2.4.0+, e-value 1e-5) to the best matched *erm* representative sequence. Similarly, metagenomes from human-impacted (Fitzpatrick and Walsh 2016) and pristine environment (Staley *et al.*^2013^) were aligned against *erm* sequences and considered a match if alignment scores resulted in e-value scores of at least 1e-5.

## RESULTS

**Figure 1.**
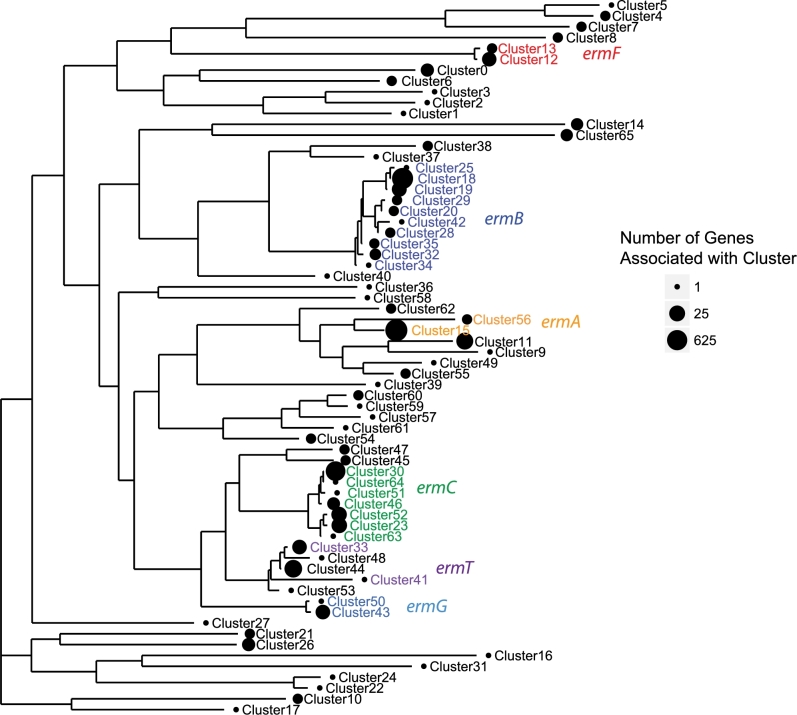
Maximum likelihood phylogenetic tree of 66 *erm* sequence clusters based on 99% nucleotide similarity of 5648 DNA sequences extracted from known *erm* genes described in existing databases. Clusters that contain gene targets from existing PCR primers (see Table 2) are highlighted in color. The relative number of sequences comprising each cluster among the 5648 DNA sequences is also shown.

A total of 5648 *erm* DNA sequences were identified from annotated genes based on sequence similarity to well-characterized *erm* genes and were clustered at 99% nucleotide similarity to identify 66 unique *erm* variant clusters. A representative sequence of each cluster was defined as the longest consensus sequence in each cluster as determined by a greedy incremental clustering algorithm (see Methods, Table 1). These representative sequences were aligned and used to construct a phylogenetic tree describing the diversity of *erm* genes (Fig. 1). Based on sequence homology, the resulting *erm* gene clusters encompass the majority of *erm* genes studied in previous literature: *ermA, ermB, ermC, ermF, ermG* and *ermT* (reviewed in Roberts *et al.*^2008^). Among the gene clusters, a cluster associated with *ermA* was the most represented in our *erm* gene database (Cluster 15, 3542 genes), followed by an *ermB* cluster (Cluster 18, 1387 genes), and then an *ermC* cluster (Cluster 30, 399 genes). These three gene clusters comprise 94% of *erm* genes and are evidence to biases in the previous characterization of *erm* genes towards specific gene variants. Beyond the three most abundant gene clusters, the next most represented cluster (Cluster 11, 50 genes) is not well-characterized (e.g. most similar to unannotated *erm* gene clusters in our database) and is most closely related to genes belonging to *Streptococcus agalactiae* strain TR7 (100% nucleotide identity). Most clusters (53 of 66) are associated with five or less gene sequences, demonstrating that much of what we know of specific *erm* gene families is based on very few characterized representatives.

**Table 1.** *Erm* gene clusters identified from 5648 *erm* sequences. For each cluster, the most representative gene is referenced by its NCBI accession number in NCBI nucleotide and protein databases.

Cluster (this study)	NCBI protein accession no.	NCBI nucleotide accession no.	Description in NCBI GenBank	Organism
Cluster 0	BAJ34818	AB601890	Erythromycin resistance protein	*Photobacterium damselae* subsp. piscicida
Cluster 1	KNF08983	LGSS01000004	rRNA (adenine-N(6)-)-methyltransferase	*Clostridium purinilyticum*
Cluster 2	AFS78141	CP003326	rRNA (adenine-N(6)-)-methyltransferase	*Clostridium acidurici* 9a
Cluster 3	ABW20380	CP000853	rRNA (adenine-N(6)-)-methyltransferase	*Alkaliphilus oremlandii* OhILAs
Cluster 4	KKS60599	LCDU01000003	rRNA (Adenine-N(6)-)-methyltransferase	Candidate division WWE3 bacterium GW2011_GWF2_42_42
Cluster 5	KKS35651	LCCU01000032	rRNA (Adenine-N(6)-)-methyltransferase	Candidate division WWE3 bacterium GW2011_GWF1_42_14
Cluster 6	AJB79756	CP010391	Hypothetical protein	*Klebsiella pneumoniae*
Cluster 7	EKD94896	AMFJ01010665	Hypothetical protein	Uncultured bacterium
Cluster 8	KKU26033	LCLY01000007	rRNA (Adenine-N(6)-)-methyltransferase	Microgenomates group bacterium GW2011_GWA2_46_16
Cluster 9	CCQ93859	CARA01000062	rRNA adenine N-6-methyltransferase	*Clostridium ultunense* Esp
Cluster 10	BAP00917	AP013353	Dimethyladenosine transferase	*Mycoplasma californicum* HAZ160_1
Cluster 11	CNJ04734	CQCN01000003	Dimethyladenosine transferase	*Streptococcus agalactiae*
Cluster 12	AAA27431	M17808.1	ermF	*Bacteroides fragilis*
Cluster 13	AAA63165	M62487.1	ermF	*Bacteroides fragilis*
Cluster 14	EEO52603	ACAB02000055.1	ermF	Bacteroides sp. D1
Cluster 15	CCJ25599	HE579073	rRNA adenine N-6-methyltransferase	*Staphylococcus aureus* subsp. aureus ST228
Cluster 16	CCX90994	CAXH010000024	Ribosomal RNA small subunit methyltransferase A	Succinatimonas sp. CAG:777
Cluster 17	EGV00599	AFXA01000001	Dimethyladenosine transferase rRNA modification enzyme	*Mycoplasma columbinum* SF7
Cluster 18	EFY03905	AEVN01000118	rRNA adenine N-6-methyltransferase	*Phascolarctobacterium succinatutens* YIT 12067
Cluster 19	ACB90575	CP001033	Erythromycin ribosome methylase	*Streptococcus pneumoniae* CGSP14
Cluster 20	EJY36237	AMBI01000188	rRNA adenine N-6-methyltransferase	*Enterococcus faecium* 510
Cluster 21	AKB11102	CP011096	16S rRNA methyltransferase	*Mycoplasma synoviae* ATCC 25204
Cluster 22	ACD66486	CP001080	Dimethyladenosine transferase	Sulfurihydrogenibium sp. YO3AOP1
Cluster 23	EIB96299	AICL01000010	rRNA methylase	*Lactobacillus salivarius* SMXD51
Cluster 24	EEP60650	ABZS01000069	Dimethyladenosine transferase	Sulfurihydrogenibium yellowstonense SS-5
Cluster 25	AFV15157	JQ655732	Erythromycin	*Clostridium perfringens*
Cluster 26	KDE45359	JFKK01000007	16S rRNA methyltransferase	*Mycoplasma hyosynoviae*
Cluster 27	ADM89794	CP002161	Putative dimethyladenosine transferase	*Candidatus Zinderia* insecticola CARI
Cluster 28	KER55751	JPHP01000035	SAM-dependent methlyltransferase	*Bacteroides fragilis*
Cluster 29	AAR27225	AY357120	N-methyltransferase	*Streptococcus pyogenes*
Cluster 30	AIU96746	KF831357	ErmC	*Staphylococcus aureus*
Cluster 31	ACG57739	CP001130	Ribosomal RNA adenine methylase transferase	Hydrogenobaculum sp. Y04AAS1
Cluster 32	AAO20906	AF205068	erm44	*Lactobacillus reuteri*
Cluster 33	AFH70049	CP003045	rRNA adenine N-6-methyltransferase	*Staphylococcus aureus* subsp. aureus 71193
Cluster 34	ACC94310	EU595407	ErmB	Uncultured Enterococcus sp.
Cluster 35	AAF86219	AF242872	ErmB	*Enterococcus faecium*
Cluster 36	CDZ75671	LM997412	rRNA adenine N-6-methyltransferase	Peptoniphilus sp. ING2-D1G
Cluster 37	EOK35943	ASEN01000042	rRNA adenine N-6-methyltransferase	*Enterococcus faecalis* EnGen0332
Cluster 38	EZX88180	JIYN01000027	rRNA adenine N-6-methyltransferase	*Staphylococcus aureus* GD2010-052
Cluster 39	CEI83544	CDGG01000001	rRNA adenine N-6-methyltransferase	*Oceanobacillus oncorhynchi*
Cluster 40	CEJ95855	LN680996	23S RNA methylase for macrolide-lincosamide-streptogramin B resistance	*Staphylococcus fleurettii*
Cluster 41	CAD32685	AJ488494	Erythromycin resistance protein	*Lactobacillus fermentum*
Cluster 42	EIY35985	AGXG01000023	rRNA adenine N-6-methyltransferase	*Bacteroides cellulosilyticus* CL02T12C19
Cluster 43	EDV04163	ABJL02000008	Hypothetical protein	*Bacteroides intestinalis* DSM 17393
Cluster 44	AHH55321	KC790462	rRNA adenine N-6-methyltransferase	*Streptococcus suis*
Cluster 45	BAB20748	AB014481	ErmGM	*Staphylococcus aureus*
Cluster 46	KAC49299	JIQI01000041	rRNA adenine N-6-methyltransferase	*Staphylococcus aureus* VET0243R
Cluster 47	CDQ41560	CCDP010000003	rRNA adenine N-6-methyltransferase	*Virgibacillus massiliensis*
Cluster 48	AGK85210	KC405064	Erythromycin ribosome methylase	*Haemophilus parasuis*
Cluster 49	BAC12877	BA000028	Erythromycin resistance protein	*Oceanobacillus iheyensis* HTE831
Cluster 50	AAC37034	L42817	rRNA methyltransferase	*Bacteroides thetaiotaomicron*
Cluster 51	EJD65709	AFSU01000133	Hypothetical protein	Bacillus sp. 916
Cluster 52	CAJ43792	AM159501	rRNA methylase	*Staphylococcus saprophyticus*
Cluster 53	EZS04927	JILJ01000152	rRNA adenine N-6-methyltransferase	*Staphylococcus aureus* VET0436R
Cluster 54	CCG55258	HE775264	Ribosomal RNA adenine methylase Erm(43)	*Staphylococcus lentus*
Cluster 55	EJY20540	AMBD01000117	rRNA adenine N-6-methyltransferase	*Enterococcus faecium* C1904
Cluster 56	CAE18145	AJ579365	rRNA methylase	*Staphylococcus sciuri*
Cluster 57	KIJ86993	JXBG01000010	SAM-dependent methlyltransferase	*Staphylococcus saprophyticus*
Cluster 58	EKB53568	AGZE01000039	Hypothetical protein	Facklamia ignava CCUG 37419
Cluster 59	CDS14986	LK392593	23S rRNA methylase	*Staphylococcus xylosus*
Cluster 60	AJK31391	KJ728534	Ribosomal RNA adenine methylase variant	*Staphylococcus xylosus*
Cluster 61	AJK31388	KJ728533	Ribosomal RNA adenine methylase	*Staphylococcus saprophyticus*
Cluster 62	KIO72601	JXLU01000090	rRNA adenine N-6-methyltransferase	*Bacillus thermoamylovorans*
Cluster 63	KKD22675	LATV01000011	SAM-dependent methlyltransferase	*Staphylococcus cohnii* subsp. cohnii
Cluster 64	EVJ59956	JBER01000028	rRNA adenine N-6-methyltransferase	*Staphylococcus aureus* GGMC6053
Cluster 65	EDU98728	ABIY02000132.1	ermF	Bacteroides coprocola DSM 17136

Next, we evaluated the diversity of bacteria carrying these *erm* genes by identifying the taxonomic origin of potential bacterial hosts associated with each *erm* gene sequence (Table 1; Fig S1, Supporting Information). In general, the majority of known *erm* gene sequences were associated with *Firmicutes* (98%), followed by *Proteobacteria* (0.6%) and *Bacterioidetes* (0.6%). While *ermF* and *ermG* genes were observed to be carried by only *Bacteriodetes*, *ermA, ermB, ermC* and *ermT* genes were associated primarily with *Firmicutes* (Fig S1, Supporting Information). Within the *Firmicutes*, *ermB* genes were associated mainly with the order *Lactobacillales*, while *ermA* and *ermT* genes were associated with members of the *Bacillales* order (Fig S2, Supporting Information). These results demonstrate a wide range of potential host diversity for *erm* genes and highlight the impact of the choice of primer gene targets selecting for or against specific host bacteria.

Historically, *erm* genes have been extensively targeted for qPCR quantification of gene abundances in the environment (Table 2), and we evaluated the ability of previously published PCR primers to detect the *erm* gene diversity described above by computationally hybridizing the primer sequences from the literature with the representative *erm* gene sequences in our database. Overall, published primer pairs were 100% similar to 25 of the representative sequences of *erm* clusters (Fig. 1). Generally, well-characterized gene clusters (e.g. containing the most known gene sequences) were observed to be associated with previous primer development. Several clusters were not associated with previously published primer targets, very likely due to the few well-characterized *erm* sequences within these clusters. Previously, observed diversity in natural samples have weak correlations with well-characterized genes (Choi *et al.*^2017^), suggesting that primer targets selected based on the most well-studied genes may not be effective in environmental samples.

**Table 2.** Previously published PCR primer and gene targets for *erm* genes.

Gene	Cluster	Primers design	Papers citing primers
*ermA*	15	Patterson *et al.*^2007^	^b^
	n/a^a^	Sutcliffe *et al.*^1996^	Martel *et al.*2003a, Jackson *et al.*^2004^, Luthje and Schwarz 2006, Garofalo *et al.*^2007^, Chenier and Juteau 2009, Zou *et al.*^2011^, Di Cesare *et al.*^2012^, Hoang *et al.*^2013^, Lerma *et al.*^2014^
	15, 56	Jensen *et al.*^1999^	Aarestrup *et al.*2000a,b, Jensen *et al.*^2002^, Petersen and Dalsgaard 2003, Whitehead and Cotta 2013
	n/a	Chen *et al.*^2007^	Sharma *et al.*^2009^, Just *et al.*^2011^, Alexander *et al.*^2011^, Wang *et al.*^2012^, Holman and Chenier 2013, Wang *et al.*^2015^, Xu *et al.*^2016^
	15, 56	Koike *et al.*^2010^	Ekizoglu *et al.*^2013^
*ermB*	n/a	Sutcliffe *et al.*^1996^	Martel *et al.*2003a,b, Cauwerts *et al.*^2007^, Ahmad *et al.*^2011^, Hoang *et al.*^2013^
	18, 19, 20, 25, 28, 29, 32, 34, 35, 42	Jensen *et al.*^1999^	De Leener *et al.*^2005^, Whitehead and Cotta 2013
	18, 19, 20, 25, 28, 29, 32, 34, 35, 42	Chen *et al.*^2007^	Sharma *et al.*^2009^, Chen *et al.*^2010^, Alexander *et al.*^2011^, Just *et al.*^2011^, Kalmokoff *et al.*^2011^, Negreanu *et al.*^2012^, Holman and Chenier 2013, Beukers *et al.*^2015^, Wang *et al.*^2015^, Sandberg and LaPara 2016, Xu *et al.*^2016^
	18, 19, 20, 25, 29, 32, 35, 42	Patterson *et al.*^2007^	Knapp *et al.*^2010^
	18, 19, 20, 25, 28, 29, 32, 34, 35, 42	Koike *et al.*^2010^	Ekizoglu *et al.*^2013^, Garder *et al.*^2014^, Joy *et al.*^2013^, Joy *et al.*^2014^, Soni *et al.*^2015^, Luby *et al.*^2016^
*ermC*	n/a	Sutcliffe *et al.*^1996^	Martel *et al.*2003b, Hoang *et al.*^2013^
	30, 46	Jensen *et al.*^1999^	Ekizoglu *et al.*^2013^, Whitehead and Cotta 2013
	23, 30, 46, 51, 52, 63, 64	Patterson *et al.*^2007^	Knapp *et al.*^2010^, Popowska *et al.*^2012^
	30, 46, 51	Koike *et al.*^2010^	Luby *et al.*^2016^
*ermF*	12, 13	Chen *et al.*^2007^	Sharma *et al.*^2009^, Chen *et al.*^2010^, Alexander *et al.*^2011^, Kalmokoff *et al.*^2011^, Negreanu *et al.*^2012^, Wang *et al.*^2012^, Hoang *et al.*^2013^, Holman and Chenier 2013, Farenfeld *et al.*^2014^, Garder *et al.*^2014^, Luby *et al.*^2016^, Xu *et al.*^2016^
	12, 13	Patterson *et al.*^2007^	Knapp *et al.*^2010^
	12, 13	Koike *et al.*^2010^	Ekizoglu *et al.*^2013^, Joy *et al.*^2013^, Joy *et al.*^2014^
	43, 50	Wang *et al.*^2005^	Wang *et al.*^2005^, Kalmokoff *et al.*^2011^
*ermG*	43, 50	Patterson *et al.*^2007^	N/A
	43, 50	Koike *et al.*^2010^	Ekizoglu *et al.*^2013^
*ermT*	33, 41	Chen *et al.*^2007^	Sharma *et al.*^2009^, Alexander *et al.*^2011^, Kalmokoff *et al.*^2011^, Wang *et al.*^2012^, Hoang *et al.*^2013^, Garder *et al.*^2014^, Wang *et al.*^2015^

a Primers did not hit any clusters.

b No relevant citing papers.

**Figure 2.**
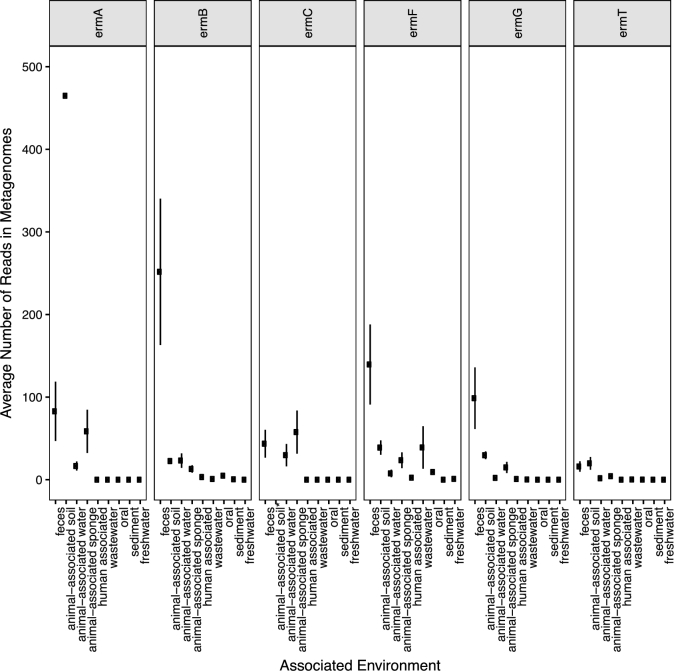
Average number of *erm* genes in metagenomes from various environments (see Table S1, Supporting Information). *For *ermC* gene, the average number of reads in animal-associated soil metagenomes was 1665 ± 659 reads.

We next evaluated the diversity of *erm* genes in 12 947 environmental metagenomes (Table S1, Supporting Information), resulting in the observation that significantly more *erm* genes are present in human-impacted environments (feces- and animal-associated soil and water) than in natural environments (Fig. 2). We also searched an additional 39 metagenomes originating from relatively pristine freshwaters along the Upper Mississippi River (Staley *et al.*^2013^, Table S1, Supporting Information), resulting in only 3 reads out of 716 million, sharing similarity (e-value < 1e-5) to *erm* genes. Combined, these results demonstrate that *erm* genes are rare in environments with minimal human impact and suggest that *erm* genes associated with feces or manure are ideal for tracking the spread and persistence of resistance through the environment. These results are also consistent with previous observations that manure contains abundant genes related to *erm* resistance and is a source of these genes into the environment (e.g. soil and water) (Chee-Sanford *et al.*^2009^; Koike *et al.*^2010^; Heuer, Schmitt and Smalla 2011; Joy *et al.*^2013^; Luby, Moorman and Soupir 2016).

Consequently, we next identified *erm genes* in manure metagenomes. We aligned *erm* gene sequences against metagenomes derived from two large manure metagenomic studies (requiring n_manure_ > 3): swine manure collected near Nashua, IA (Luby, Moorman and Soupir 2016) and cattle manure from a previously published study (Noyes *et al.*^2016^). These manure metagenomes were strategically selected based on the number of biological replicates and sequencing depth. Three *erm* clusters comprised 46% and 45% of the total abundance of *erm* genes in swine and cattle manure, respectively (Table S1, Supporting Information). The genes associated with these most abundant clusters differed between swine and cattle manures. In swine metagenomes, sequences associated with the *ermB* gene cluster (Fig. 1, sharing 93%–99% similarity) captured 26% of all *erm* sequences, followed by *ermG-*associated sequences capturing 11% and *ermA-*associated sequences capturing 9%. In cattle metagenomes, sequences associated with *ermF* represented 23.5% of all *erm* abundances, followed by sequences associated with *ermG* capturing 12.4% and sequences associated with *ermB* capturing 9%.

**Figure 3.**
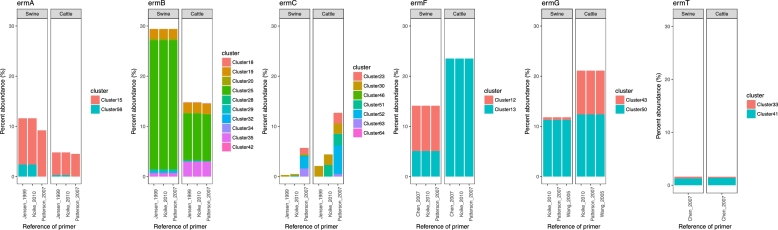
Abundance of DNA sequences homologous to *erm* gene PCR primers as a percent of total *erm* abundance in swine and cattle metagenomes.

Only a subset of *erm* genes detected in manure are targeted by existing primer sets. Overall, a total of 25 out of the 66 *erm* clusters (40%) could be computationally detected with known primers (Table 1, Supporting Information), and these genes also encompass much of the total *erm* abundances observed in manure metagenomes. Collectively, if all primers were used, 74% and 85% of the total *erm* gene sequence diversity observed in swine and cattle metagenomes, respectively, could be detected, suggesting good coverage of these genes for PCR or qPCR assays. Specifically, in swine manure metagenomes, *ermB* primers could detect 29% of *erm* sequences, followed by *ermF* primers capturing 14% and *ermG* primers capturing 12% (Fig. 3). In cattle, *ermF* primers are the most effective, capturing 30% of *erm* sequences, followed by 21% with *ermG* primers, and 15% with *ermB* primers. Consequently, depending on the environmental sample in a study, in this case swine versus cattle manure, the choice of *erm* gene targets can significantly alter *erm* abundance estimations. For example, in swine manure, two times more *erm* gene abundance would be estimated if *ermB* primers were used instead of *ermF* primers. Even within the same gene clade, different primers could result in significant differences in abundance estimations, and this result is observed especially for *ermC* primers where a near two-fold difference in abundance estimations would result based on selection of primers from Patterson *et al.* (2007) versus Jensen, Frimodt-Moller and Aarestrup (1999). The selection of Patterson primers would result in the detection of genes from up to seven *erm* gene clusters over the two to three gene cluster detected with Jensen, Frimodt-Moller and Aarestrup (1999) or Koike *et al.* (2010) primers. Similar results are noted in the cattle manure, where *ermC* primers designed by Patterson capture 13% of the total abundance of *erm* sequences in the metagenomes, while Koike and Jensen primers only capture 4.4% and 2.1%, respectively. These results emphasize that the targeting of a specific *erm* gene, even within closely related gene variants, can significantly alter estimations of associated resistance in manures.

Thus, overall, for swine manure, the most effective gene target based on abundance in swine metagenomes (26% of *erm* genes) originates from an *ermB* cluster (Cluster 25) and is associated with *Clostridium perfringens*. The next most abundant *ermB* cluster in swine (Cluster 19, most similar to a gene in *Streptococcus pneumoniae CGSP14*) represented only 2% of *erm* abundances. These results indicate that while *ermB* primers can target multiple strains (Fig. S1 and S2, Supporting Information), in these swine metagenomes, it is one gene cluster that specifically dominates. This gene cluster is also abundant in cattle manure metagenomes, though comprising less of total *erm* gene abundance (9%). Within our *erm* gene database, this particular sequence cluster is represented by a single gene representative and shares 100% similarity to experimental *Clostridium acetobutylicum* strains in the NCBI non-redundant gene database (mutant HQ683763.1 and clone HQ25744.1). The overall lack of similar homologous genes in NCBI nr suggests that this specific *ermB* gene is abundant in manures but is a gene for which we have few sequenced representatives. We identified this gene during our exploration of the effectiveness of current primers on manure metagenomes, and our observations suggest that this gene would benefit from further study given its prevalence.

## DISCUSSION

Over the past 20 years, an abundance of literature has been published quantifying macrolide resistance in agricultural landscapes using qPCR approaches. However, these previous studies often use primers for *erm* genes designed in only a handful of publications (Table 2). Our study found that current published primer sets, used on their own, are effective at capturing only a subset of the *erm* diversity in manure samples. For example, if only one primer set were used, less than one-third of *erm* genes would be detected. To increase our ability to detect *erm* genes in agricultural systems, we identified the most abundant *erm* clusters in both swine and cattle manures, identifying the best gene targets for future studies. These genes and their associated primers are recommended for high-throughput qPCR assays that can scale the detection and quantification of these genes for antibiotic gene surveillance.

In all amplicon assays, quantifying environmental abundances of gene targets is limited by the effectiveness of primer design. The results presented here emphasize that estimates of abundances of a gene of interest cannot simply be based on primers to genes that have previously been successfully detected. Rather, genes appropriate for antibiotic gene surveillance should be indicative of the spread of resistance (e.g. originate from manure but lacking from pristine environments), representative of diverse hosts (especially those with clinical risks) and accurately represent gene abundances in environmental samples. Our specific effort targeted the *erm* gene and evaluated the effectiveness of previously published primers sets. The increasing availability of metagenomes makes these evaluations possible, as demonstrated in this study. Although metagenomic sequencing advances will continue to provide powerful tools to understand the broad diversity of resistance in environments, metagenomes are limited by both detection rate and resolution. Short read lengths, the difficulty of assembling many resistance genes (because of their common association with mobile elements containing repeated sequences) and their presence in multiple bacterial hosts challenges the detection of resistance genes using metagenomics. Going forward, high-throughput amplicon assays with strategic gene targets and primer designs are a complementary alternative to help fill these gaps and help us understand the movement of resistance genes among complex environments.

## Supplementary Material

Supplementary material
