# Supplementary figures and images for: Practical implications of erythromycin resistance gene diversity on surveillance and monitoring of resistance
Source: FEMS Microbiol Ecol. 2018 Jan 15;94(4):fiy006. doi: 10.1093/femsec/fiy006 (PMC5939627; doi:10.1093/femsec/fiy006)

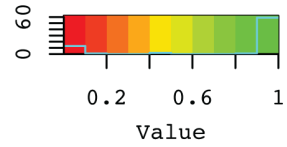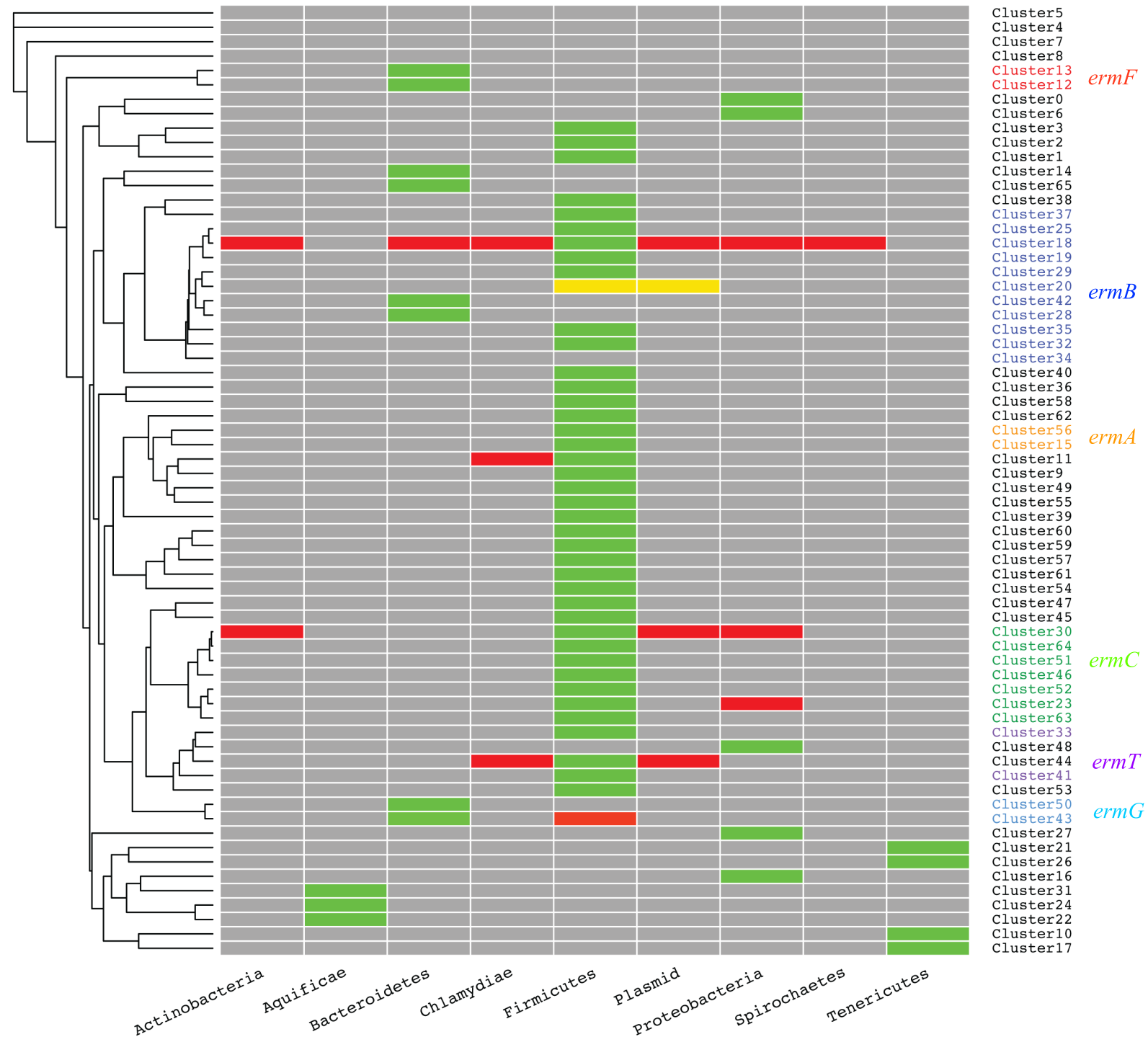

Supplement: Supplementary material [file fiy006_supp.zip › supp-fig1-Phylogeny-all.pdf]

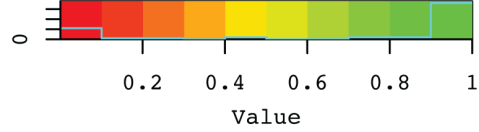

# Distribution of Orders within Firmicutes

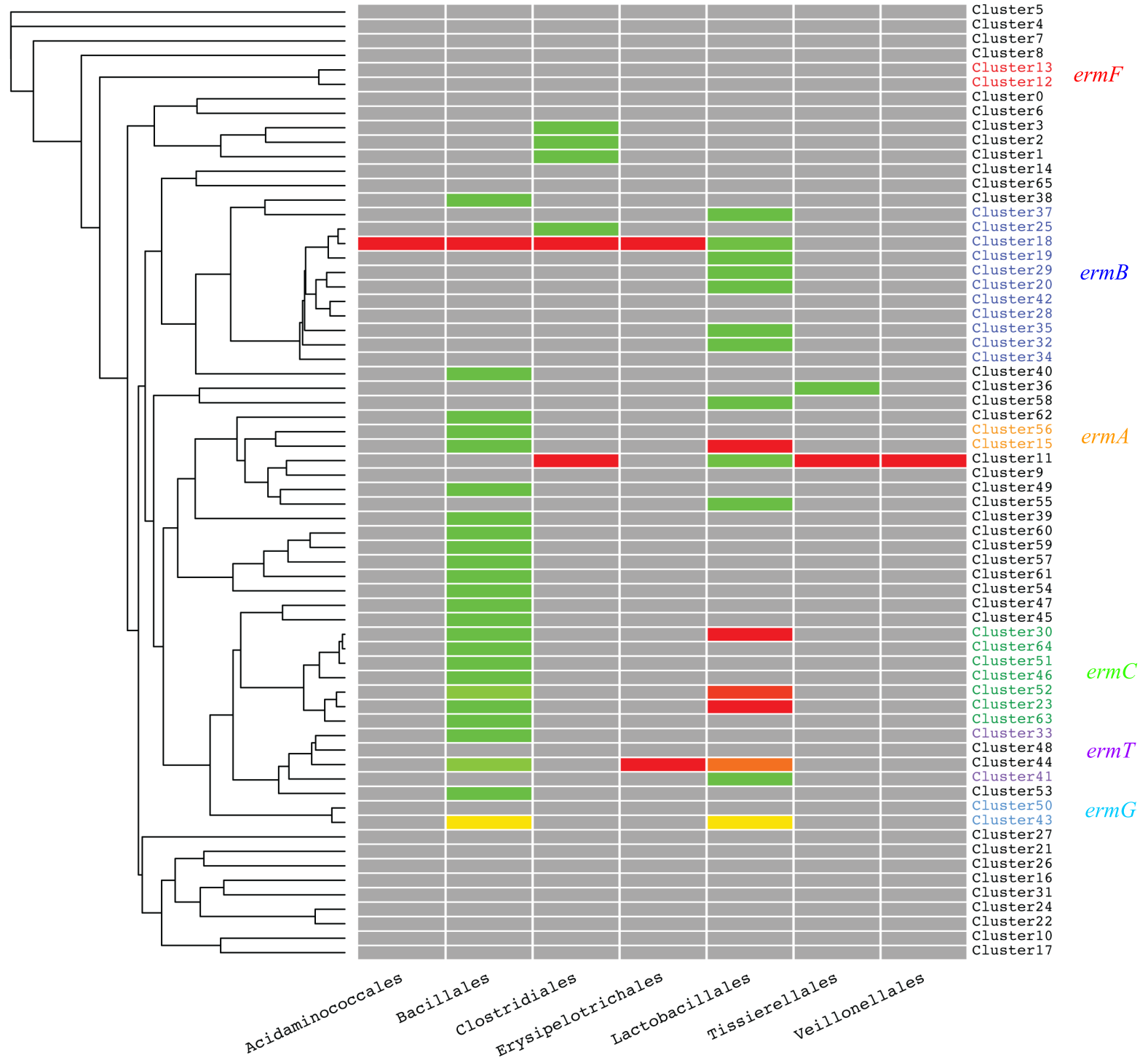

Supplement: Supplementary material [file fiy006_supp.zip › supp-fig2-Phylogeny-Firmicute.pdf]
